# NEMiD: A Web-Based Curated Microbial Diversity Database with Geo-Based Plotting

**DOI:** 10.1371/journal.pone.0094088

**Published:** 2014-04-08

**Authors:** Kaushik Bhattacharjee, Santa Ram Joshi

**Affiliations:** Microbiology Laboratory, Department of Biotechnology and Bioinformatics, North-Eastern Hill University, Shillong, Meghalaya, India; Louisiana State University and A & M College, United States of America

## Abstract

The majority of the Earth's microbes remain unknown, and that their potential utility cannot be exploited until they are discovered and characterized. They provide wide scope for the development of new strains as well as biotechnological uses. The documentation and bioprospection of microorganisms carry enormous significance considering their relevance to human welfare. This calls for an urgent need to develop a database with emphasis on the microbial diversity of the largest untapped reservoirs in the biosphere. The data annotated in the North-East India Microbial database (NEMiD) were obtained by the isolation and characterization of microbes from different parts of the Eastern Himalayan region. The database was constructed as a relational database management system (RDBMS) for data storage in MySQL in the back-end on a Linux server and implemented in an Apache/PHP environment. This database provides a base for understanding the soil microbial diversity pattern in this megabiodiversity hotspot and indicates the distribution patterns of various organisms along with identification. The NEMiD database is freely available at www.mblabnehu.info/nemid/.

## Introduction

The proliferation of biological databases and the easy access enabled by the Internet has a beneficial impact on the biological sciences and has transformed the way research is conducted [1]. Anthropogenic interventions have caused alterations in the diversity of microorganisms and in the biodiversity of higher organisms. The unseen microbial resource, encompassing a diverse spectrum of microorganisms, deserves immediate attention in terms of documentation and bioprospection. This diversity can be of immense value to biotechnology for the exploration and documentation of new genes. The microbial diversity pattern can also be used to monitor environmental changes. Efforts aimed at the conservation of higher organisms can be correlated and modeled in microorganisms for understanding the evolutionary processes and biological interactions that sustain natural ecosystems. As biotic components, microorganisms play vital role in the maintenance of Earth's ecosystems. However, they remain largely unused as they are not documented properly. They can be tapped for use in biotechnological applications such as the development of pharmaceuticals, the synthesis of new enzymes and chemicals, and in even carrying out novel processes [2]. Knowledge of microbial diversity is scarce in spite of their importance to human welfare. As the enormity of microbial diversity becomes apparent, the dilemma of how to preserve microorganisms and their gene pools asserts itself ever more forcibly. Although there have been swift rise in the strategies recommended for conservation of biodiversity but none seems to consider microorganisms. The documentation and bioprospection of microorganisms carry enormous significance considering their potential for human welfare. There is a perceived need to develop databases with emphasis on the microbial diversity of the largest untapped reservoirs in the biosphere. The occurrence of life forms in a region can be linked to many ecological factors and the altitudinal gradient is well known as the decisive factor [3], [4], [5]. North-East India, located in the Eastern Himalayan region, one of the megabiodiversity hotspots of the world, has pristine natural ecosystems which offer scope for the bioprospection of novel organisms hitherto unknown to scientific world [6]. Exploration of microbial diversity from diverse ecological niches of the Indo-Burma biodiversity hotspot holds promise for the isolation of biotechnologically significant strains and even novel species [7]. The present database is aimed at characterizing and documenting microbes from the pristine gradients of the Eastern Himalayan range. The most defined characteristics of this region is that it harbors diverse types of habitats comprising gradients of altitudinal variation, temperature and soil offering wide scope for exploration and characterization of the yet unexplored microbial diversity of the region. Microorganisms are rapidly disappearing from the Earth due to anthropogenic disturbances and approaches for proper documentation of microbes would be instrumental in protection and bioprospection of microbial biodiversity.

## Materials and Methods

### 1. Data Collection and curation/organization

**Figure 1:**
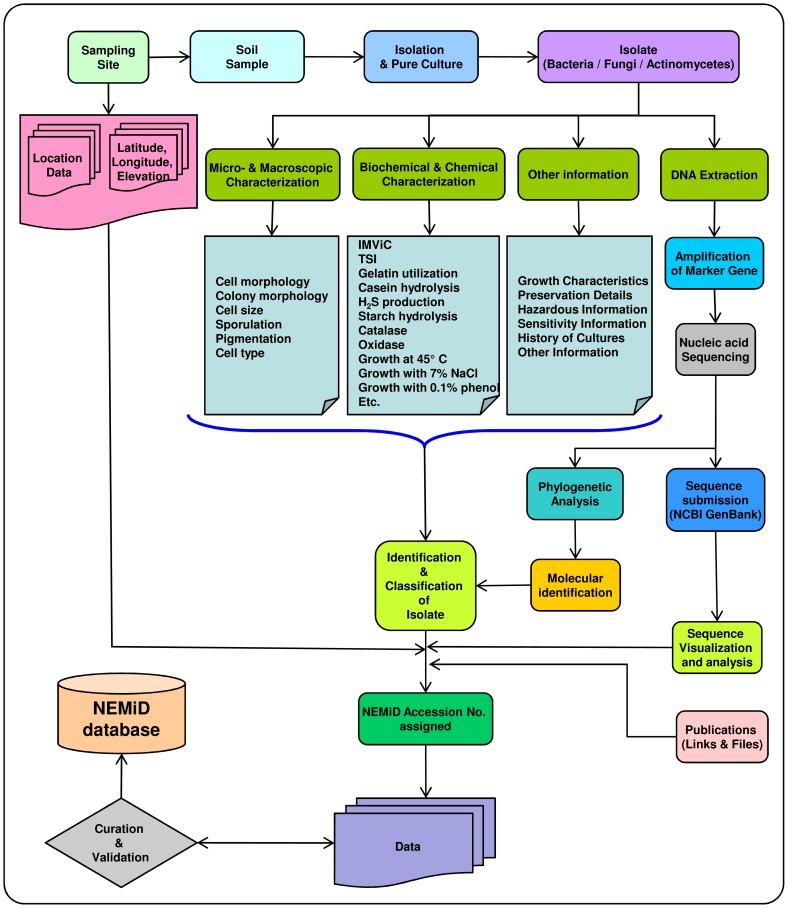
Flow-chart depicting data generation & curation in NEMiD.

There were no specific permissions required for collecting soil samples from different parts of the sampled region within the territory of India. This study did not involve any endangered flora or fauna of the region nor any protected areas; rather, it involved the exploration and finding of possible new and/or already known microbes of the region from the soil samples from within the boundaries of India. The data annotated in NEMiD were obtained by isolating microbes from soil samples collected from different parts of the region, and characterizing the isolates using microscopic, biochemical and molecular approaches. The strains identified and included in the database are deposited in different microbial culture collection centers namely Microbial Repository Centre, Institute of Bioresources and Sustainable Development, Imphal (India), Microbial Type Culture Collection and Gene Bank, Institute of Microbial Technology (India) and National Bureau of Agriculturally Important Microorganisms (India).The same strains are also preserved in the departmental repository of the parent university. An experimental record of an isolated microbe can be thought of as an independent data unit of the NEMiD, which includes a discrete set of parameters (colony and cell characteristics, growth characteristics, preservation details, history of cultures, etc.) (Figure 1). The strains annotated in the database can be supplied for non-commercial usage provided the user meets the required conditions.

### 2. Database design and architecture

**Figure 2:**
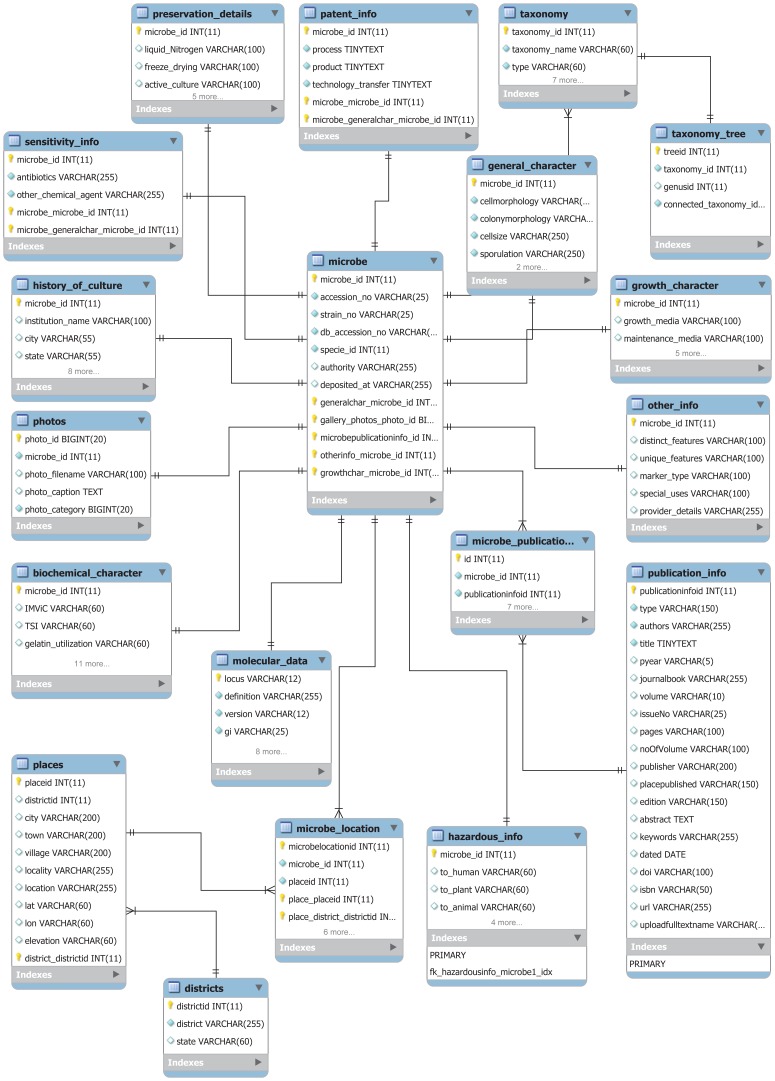
Entity relationship diagram (ERD) of the NEMiD model as a MySQL database. Boxes show different tables (titles listed on top of the individual tables). Foreign keys between tables are shown. Some details of the model have been ignored to reduce the complexity of the diagram.

**Figure 3:**
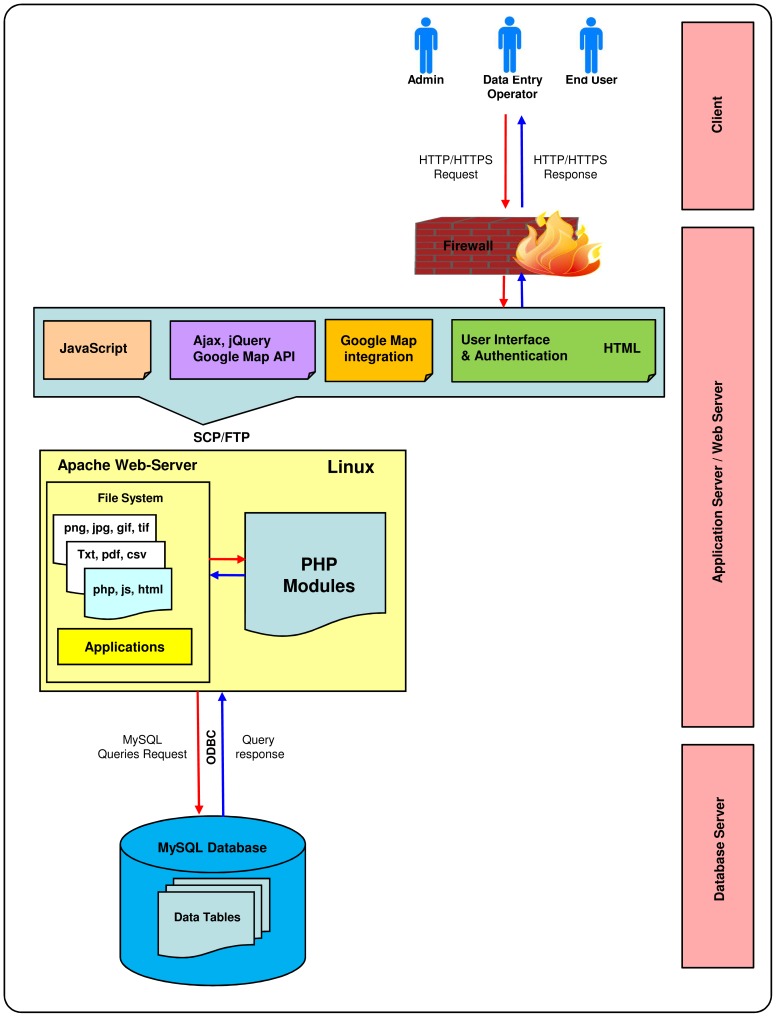
Schematic representation of the data mining flow of the NEMiD database. The user sends an HTTP request to the Apache Web Server, which is then relayed to the PHP module where a job is created. When the job is complete, the results are formatted, returned through the Web Server and accessed via the web interface. Authentication is only required for data entry and editing.

The main component of the architecture is the central data warehouse used to store the contents of the databases to provide integrated access based on Linux-Apache-MySQL-PHP (LAMP) Server technology. The database was constructed as a relational database management system (RDBMS) for data storage in MySQL 5.5.32 (http://www.mysql.com/) in the back-end on a Linux server (Figure 2). Queries to the database are implemented in PHP scripts running in an Apache/PHP environment. “Phylogenetic Information” is modeled on a hierarchical database module (Figure S1) to nullify any wrong data entry or redundancy, and a looping-through database query is followed to overcome issues such as same values in both the tables (e.g., for *Streptomyces griseoruber*, both the Phylum and Class are Actinobacteria). A further benefit of this architecture is that the location data table (latitude, longitude, and altitude) can be imported and used in GIS packages like ArcGIS to develop a ‘shape file’ for a geo-informatics-based study of the area. NEMiD is designed to plot the collection locality of a microbial isolate with the help of Google Maps, when the database has geographic coordinates for the particular isolate. The page for that isolate will show the map with a tag pinpointing the specific locality, and this map can be zoomed in and out and dragged around by using the mouse. This is accomplished by implementing the Google Maps API 3.0 (https://developers.google.com/maps/) (Figure 3).

The relational architecture of NEMiD ensures data integrity and future expandability. In addition, NEMiD makes use of custom-designed lookup tables that ensures rapid responses to search queries. To assist in modeling efforts, a NEMiD Accession Number is created for each isolate entered in the database (Table S1). The database structure was designed to be modular, to avoid unnecessary redundancy and to allow fast queries. The database schema conforms to a set of functional dependencies designed to avoid unnecessary data duplication. Functional dependencies are considered standard practice in establishing good database designs [8]. The data inserted into this central data store can have its origin in different kinds of sources. Once the data are available in the central data warehouse, uniform access via a standardized query language is provided. The contents of the central data warehouse are then made available for interactive querying using a standard three tier approaches consisting of the central data store as the back-end, a web server capable to dynamically create hyper text markup language (HTML) pages as the middle tier, and web browsers as light-weight clients (Figure 3). In addition to data access via web browsers, the query language of the underlying RDBMS can be used directly to access data at different integration levels by the database administrator.

### 3. Web interface

NEMiD provides an intuitive mouse-based interactive web-based graphical user-friendly interface (GUI) to search for microorganisms by Genus or species. These interfaces allow a user to display and browse taxonomic information, biochemical characteristics, growth characteristics, preservation details, hazardous information, sensitivity information, geo-location, etc. NEMiD is implemented on an open-source software platform and PHP serves as the common gateway interface (CGI) to the relational database. The web interface was developed using PHP 5.2.3 as a web front-end that dynamically generates HTML pages and is hosted on an Apache 2.0 web server running CentOS 6.2. In the NEMiD web interface, the Asynchronous JavaScript and XML (Ajax) package jQuery are implemented throughout the site to improve the user experience (UX) [9] in search results and for the normalization of the cross-browser JavaScript functionality in web applications. The package is designed to run on multiple platforms and has been tested successfully with older versions of PHP and alternate web servers such as IIS and Apache 1.3.

### 4. Curation

The information contained in NEMiD is manually entered using a web interface that involves data checking and validation. Automated search and manual curation are combined to improve the quality of the database [10]. Manual curation of the NEMiD is carried out for each item present on the displayed pages, and the placement of the taxa in the hierarchical taxonomy is based upon the phylogenetic analysis of the molecular data in reference sequence databases such as NCBI GenBank or/and the EzBioCloud database [11].

## Results and Discussion

### 1. Database Description

The NEMiD web portal is available at http://mblabnehu.info/nemid. It is designed to answer primary and integrative analysis questions frequently asked by microbiologists and biotechnologists. The current version provides different types of information extracted from more than 140 study sites profiling more than 229 microbial isolates, with 229 16S rRNA partial sequences. Cross-links to other relevant databases are present, for access to the molecular properties of sequenced gene(s) from NCBI GenBank, and related literature from NCBI PubMed and other journal web-pages. The database is available without any login requirement to facilitate easy access without restrictions. Users can also download all the publications related to the data from the web page using the “Full text paper-Download” function. The data inserted into this central data store can originate from different kinds of sources. Once the data are available in the central data warehouse, uniform access via a standardized query language is provided. The contents of the central data warehouse can then be made available for interactive querying using a standard three-tier approach consisting of the central data store as the back-end, a web server capable of dynamically creating HTML pages as the middle tier, and web browsers as lightweight clients. In addition to data access via web browsers, the query language of the underlying RDBMS can be used directly to access data on different integration levels by database administrator (Figure 3). It was necessary to create an efficient data updating system so that users will be able to add new entries to maintain the usefulness of the database in the long run. For this, a system that facilitates data updating via a necessary ‘User's login’ option has been developed. This provides the interface to add data in prescribed field formats. Manual curation and validation of the data is performed by the curator of the database and are stored in the main data storage table through a PHP script.

### 2. Testing

The NEMiD database was tested by using data generated from the study areas. The data was entered in all the PHP modules and validated at two stages: at the web-page entry stage and at the PHP MyAdmin database stage. These processes were carried out to ensure the quality of the data in the database.

### 3. Web interface

**Figure 4:**
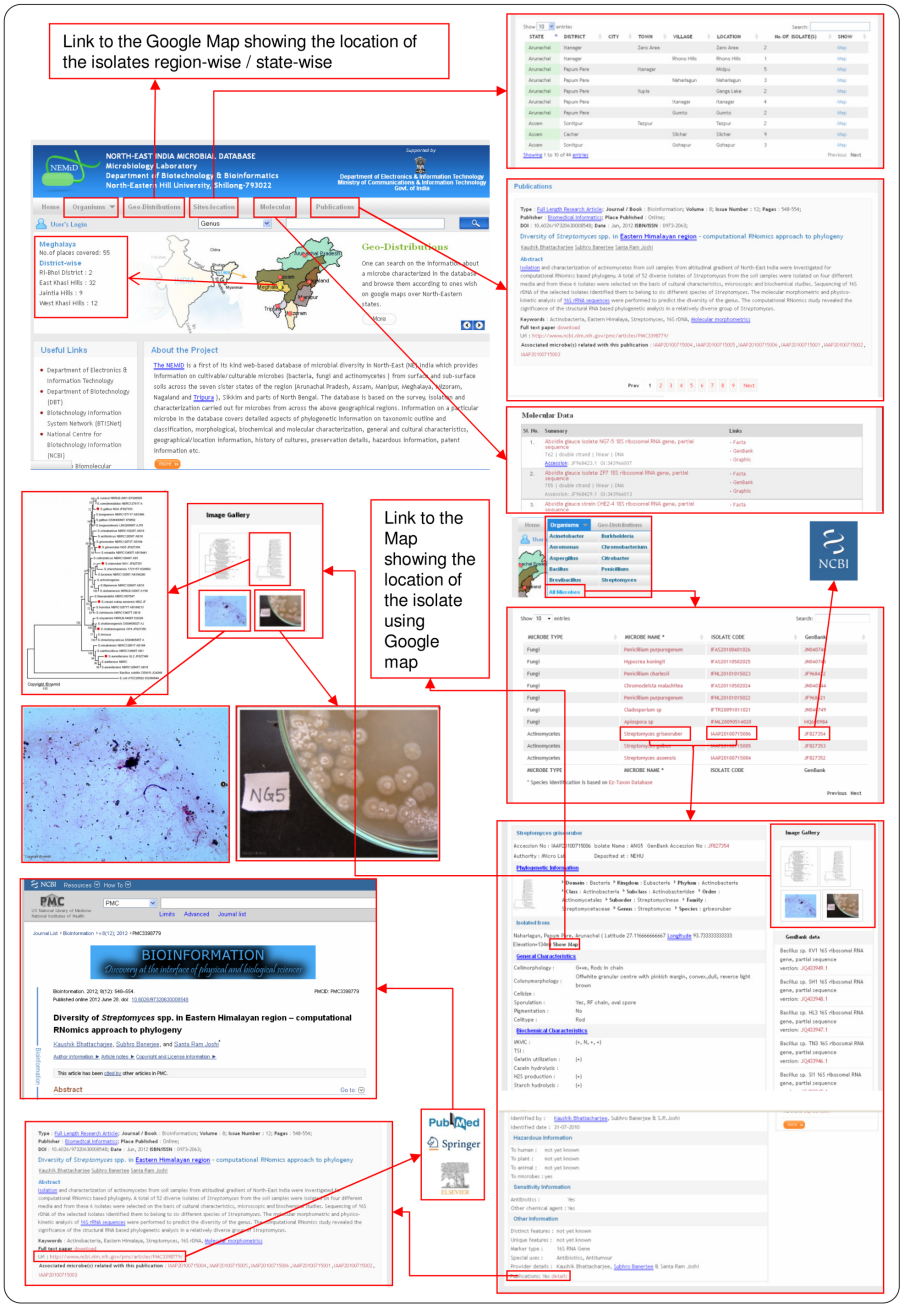
Screenshot and example of output results of the NEMiD web interface.

The NEMiD web interface is designed to be simple and user-friendly for querying different types of information including details of the organism, molecular data, publications, etc. The user can select the microbe of interest from the list of microbes in the ‘Organisms’ menu. Alternatively, the user can use the quick search option present in the web interface and obtain information on the relevant Genus or Species. The Results page of the organism search also includes hyperlinks to images of the isolate (i.e., Image Gallery), publication data, GenBank sequence, and a location map using the ‘Show Map’ to access detailed data. Users can also search microbial geo-distributions and site locations in the corresponding sections. The geo-distribution search yields the location-specific distribution of a particular organism and the site location search provides a location-wise distribution of microbes. A printing option for the displayed page is also present in the web portal. The web portal has been optimized for viewing using Netscape Navigator under both Linux and MS Windows operating systems; however it has also been successfully tested in different internet browsers like IE 8.0, Firefox, Opera, Google Chrome, etc. for data exploration and visualization. The ‘Contact Us’ tab located at the bottom of the page is for any query or required assistance (Figure 4).

### 4. Comparison to Other Related Databases

The availability of biodiversity data can be of a major relevance at a time of global habitat loss [12]. Several existing databases and web portals have been developed for bio-diversity data, but do not focus on microbiological data (e.g. BODHI [13], HimFlorIS [14], GRAMENE [15]). Very few computational resources have been developed in the area of microbial diversity and related fields. These databases are more specialized and have focused on a specific microbe or niche such as fungal diversity and evolution (PHYMYCO-DB [16]), extremophiles (ExtremeDB [17]), human oral microbiome (HOMD [18]), or pathogenic bacteria (PATRIC [19]). Some databases have focused mainly on the molecular data of microbes, such as the CORE [20], LAMP [21], PolysacDB [22], MiST 2.2 [23], EzTaxon-e [11], RDP [24], and GenBank databases. Despite the critical value of microbial diversity in our biosphere, no effort has been made to compile their unique properties on a single platform. NEMiD provides a single platform that deals with microbial diversity, with information on the characteristics, molecular data, and location of the soil microbes of the Eastern Himalayan range.

## Conclusion and Future Prospects

NEMiD currently contains 229 entries of bacteria, fungi, and actinomycetes along with research articles on database-related data. New information can be incorporated or corresponding new databases can be cross-linked to NEMiD to provide a comprehensive description of microbial diversity. Such scientific investigations are of great significance to several domains of research such as biology, microbiology, biotechnology, and biochemistry and require access to a useful repository of information, followed by its maintenance for further use. Microbiologists and biotechnologists can utilize NEMiD to understand the microbial diversity pattern of the megabiodiversity region and to assist in identification of microbes. Depending on the use of NEMiD by researchers, and based on suggestions from them, other microbes from other different sources other than soil might be added in the future. Future work will mainly involve extending the coverage of NEMiD to include more microbial data, as well as data from new locations of the regions. There is a continuous effort and plan to include data from other sampling sites of the region as there are no web-sites/databases available for this region so far other than the present one. Other work will include adding geo-informatics-based diversity analyses to the database and creating FTP pages to allow data download. Continuous updates are planned for the database with new data generation and through collaboration with users and researchers of the region who are invited to report new microbial isolates from the region. To meet this need, provision has been made for inclusion of primary data generated by other researchers for this region. To facilitate such cooperation, forms for easy submission will be made available (on request). The developed database is dedicated to microorganisms from a megabiodiversity region, and is expected to help in understanding the microbial diversity pattern of the region as well as its relationship to climate change studies. NEMiD will facilitate easy access to the microbial diversity data of the region and would open up new avenues for microbiological databases in basic and applied research.

## Supporting Information

Figure S1 **Hierarchical module of the “Phylogenetic Information” model.**
(TIF)

Table S1 **NEMiD database accession number schema.**
(DOC)
