# Supplementary figures and images for: NEMiD: A Web-Based Curated Microbial Diversity Database with Geo-Based Plotting
Source: PLoS One. 2014 Apr 8;9(4):e94088. doi: 10.1371/journal.pone.0094088 (PMC3979743; doi:10.1371/journal.pone.0094088)

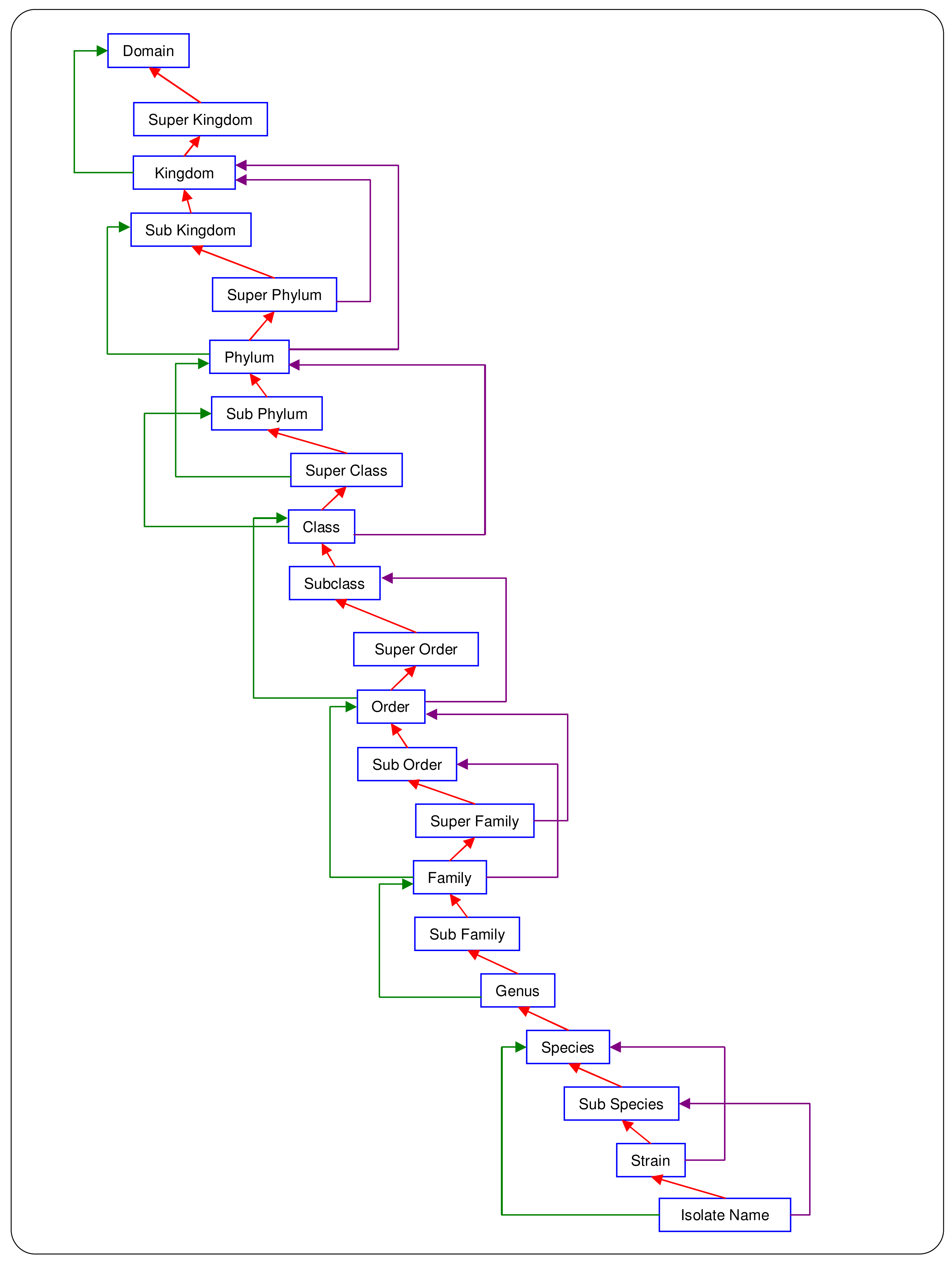

Supplement: Figure S1 — Hierarchical module of the “Phylogenetic Information” model. (TIF) [file pone.0094088.s001.tif]
