# Supplementary material for: NEMiD: A Web-Based Curated Microbial Diversity Database with Geo-Based Plotting
Source: PLoS One. 2014 Apr 8;9(4):e94088. doi: 10.1371/journal.pone.0094088 (PMC3979743; doi:10.1371/journal.pone.0094088)
Supplement: Table S1 — NEMiD database accession number schema. (DOC) [file pone.0094088.s002.doc]

Table S1: NEMiD database accession number schema.

| **Position in Acc. no.^$^** | **Options** | **Meaning** | **Type** |
| --- | --- | --- | --- |
| 1 | I | Isolate | Alphabet |
|  | S | Strain |  |
| 2 | B | Bacteria | Alphabet |
|  | F | Fungi |  |
|  | A | Actinomycetes |  |
| 3-4 | ML | Meghalaya | Alphabet |
|  | AS | Assam |  |
|  | MZ | Mizoram |  |
|  | MN | Manipur |  |
|  | AP | Arunachal Pradesh |  |
|  | TP | Tripura |  |
|  | SK | Sikkim |  |
|  | NG | Nagaland |  |
| 5-8 | NNNN, e.g, 2011 | Year of isolation | Numerical |
| 9-10 | NN, e.g, 05 | Month of isolation | Numerical |
| 11-12 | NN, e.g, 12 | Date of isolation | Numerical |
| 13-16 | NNN, e.g, 007 | Number | Numerical |

^$^: Accession number
